# Temperature-Dependent Evolution of Coke Structure and Growth Mechanisms of SiC Whiskers Synthesized from Coke and Silicon Powder Under Graphite-Burial Conditions

**DOI:** 10.3390/ma19173785

**Published:** 2026-09-06

**Authors:** Pengcheng Jiang, Huidong Tang, Xin Xiong, Wenting Wang, Xinwei Ou, Kai Chen, Zhiwen Li, Kang Long, Yongming Kang, Zhi Wu

**Affiliations:** School of Materials Science and Engineering, Hunan Institute of Technology, Hengyang 421002, China; tanghuidong@hnit.edu.cn (H.T.);

**Keywords:** SiC whiskers, coke, graphite-burial treatment, microstructural evolution, growth mechanism

## Abstract

Silicon carbide (SiC) whiskers are effective reinforcements for carbon-containing refractories; however, their application is restricted by the inherently low reactivity of graphite, which is commonly used as an internal carbon source. In this study, low-cost coke and silicon powder were used as the carbon and silicon sources to synthesize SiC whiskers via a graphite-burial heat treatment process. The structural evolution of coke and the reaction behavior of the coke–Si system at different temperatures were systematically characterized. The results reveal that coke exhibits a TG-DSC mass loss of 16.01% and undergoes progressive structural rearrangement and enhanced short-range ordering at 1200–1400 °C while retaining a predominantly turbostratic carbon structure and morphological stability. At 1100–1200 °C, SiC whiskers form predominantly within pores, and this distribution is consistent with a gas-phase-assisted VS-type growth pathway; the normalized crystalline SiC fractions remain relatively low (~4–11%). A pronounced enhancement of SiC formation occurs at 1300 °C, where the crystalline Si phase becomes undetectable by XRD and abundant SiC whiskers spread from localized pore regions to the matrix surface. The enhanced whisker formation at 1300–1400 °C is attributed to accelerated reaction and mass-transfer processes at elevated temperatures. The synthesized whiskers consist of crystalline β-SiC, and those formed at 1400 °C exhibit high-density stacking faults and pronounced radial coarsening, with representative diameters of approximately 35–80 nm in the TEM images. The structural evolution of coke from a defect-rich state toward a more ordered turbostratic structure occurs concurrently with the temperature-dependent evolution of SiC nucleation and growth and may contribute to the availability of reaction sites and transport pathways, establishing coke as a promising carbon source for the in-situ synthesis of SiC whiskers in refractory applications.

## 1. Introduction

Silicon carbide (SiC) whiskers are widely used as one-dimensional ceramic reinforcements because of their high strength, excellent thermal conductivity, low thermal expansion, and good thermal and chemical stability [1,2,3]. Their high aspect ratio enables effective load transfer and activates toughening mechanisms such as crack bridging, crack deflection, and whisker pull-out. In carbon-containing refractories, the introduction or in situ formation of SiC whiskers can improve matrix bonding, inhibit crack propagation, and enhance mechanical strength, thermal-shock resistance, oxidation resistance, and slag-corrosion resistance [4,5,6].

Graphite is commonly used as the principal carbonaceous component in carbon-containing refractories because of its excellent thermal stability, low wettability by molten slag, and high thermal-shock resistance [7,8,9]. However, its highly ordered sp^2^-bonded structure contains relatively few chemically active sites. Most reactive carbon atoms are concentrated at structural defects, exposed edges, and crystallite boundaries. Consequently, the high degree of graphitization of graphite limits its reactivity toward Si-containing species and may hinder the nucleation and formation of SiC whiskers [10]. Previous studies have shown that introducing defects into graphite through oxidation or expansion treatments can increase its reactivity and promote ceramic-phase bond formation. Zhu et al. [11] found that converting graphite into expanded graphite through oxidation disrupted its structural order and increased its reactivity, thereby facilitating the formation of non-oxide ceramic phases in MgO–C refractories. Wang et al. [12] employed expanded graphite as a carbon source in Al_2_O_3_-C refractories and observed that it similarly promoted the formation of non-oxide ceramic phases, confirming that disrupting the ordered graphitic structure effectively increased the reactivity of carbon sources. Zhang et al. [13] reviewed various graphite surface modification strategies, including surface oxidation, surfactant treatment, and coating methods, which are generally intended to modify the surface structure and properties of graphite. In essence, these modification approaches share a common underlying principle: increasing the structural disorder and defect density of graphite can lower kinetic barriers for SiC formation, making the carbon source more reactive toward other species at lower temperatures.

SiC synthesis is governed by multiple factors, including temperature, atmosphere, and the nature of the carbon and silicon sources [14]. Among these, the structural characteristics of the carbon source are particularly important because they affect the availability of active sites for nucleation and the stability of carbon supply for whisker growth. Li et al. [15] synthesized core–shell-structured SiC nanowires using graphite powder as the carbon source via a carbothermal reduction process and found that the amount of graphite powder significantly affected the nanowire morphology. Yang et al. [16] employed glassy carbon as the carbon source in a catalyst-free carbothermal reduction process to synthesize β-SiC nanowires. The amorphous structure of glassy carbon provided abundant reactive sites, which promoted the preferential growth of SiC and the formation of single-crystalline nanowires. Tian et al. [17] used coconut shells as the carbon source to synthesize SiC nanowires via chemical vapor deposition. The high reactivity and porous structure of the coconut shells facilitated gas–solid reactions and reduced the SiC synthesis temperature to 1150 °C. These findings suggest that defects and disordered domains in carbon sources provide reactive sites for SiC nucleation, while a relatively ordered carbon structure ensures a stable and continuous carbon supply for whisker growth.

Coke is an inexpensive and widely available carbon material composed mainly of imperfectly stacked aromatic carbon microcrystallites. Compared with highly crystalline graphite, coke generally contains more structural defects, exposed edges, disordered domains, and interconnected pores, which can provide reactive sites and transport channels during SiC formation [18]. Coke has been recognized as an effective carbon source for SiC whisker formation. Wang et al. [19] reported that coke or anthracite, whose reactivity is higher than that of graphite, accelerated the formation of thick β-SiC whiskers in SiC refractories when used as the carbon source embedded with Si powder. Narciso-Romero et al. [20] studied the carbothermal reduction of silica using four petroleum cokes and three metallurgical cokes. They found that the SiC yield at 1400–1500 °C is affected by the degree of anisotropy and the size and crystallographic order of the anisotropic components in coke, with larger, more ordered anisotropic structures giving higher SiC yields. This higher reactivity of coke compared to graphite has been attributed to its less ordered carbon structure, which provides more active sites for reaction with Si-containing species [21]. Furthermore, the structure and properties of the carbon source significantly influence the formation and subsequent transformation behavior of SiC. Jayakumari et al. [22] investigated the influence of different carbon sources (charcoal, coal, and petroleum coke) on the transformation behavior of SiC polytypes and found that petroleum coke hindered the β-to-α SiC transformation, with the low porosity of petroleum coke limiting the availability of reactive sites and thus affecting the reaction activity. These findings highlight the potential of coke as a cost-effective and reactive carbon source for in-situ SiC whisker formation in carbon-containing refractories.

In this work, SiC whiskers were synthesized from low-cost coke and Si powder through a graphite-burial heat treatment. The structural evolution of coke at 600–1400 °C and the phase and microstructural evolution of the coke–Si products at 1000–1400 °C were systematically characterized. This study aims to clarify the temperature-dependent formation behavior of SiC whiskers and evaluate the feasibility of using coke as an economical internal carbon source for carbon-containing refractories.

## 2. Experimental Section

### 2.1. Raw Materials

Petroleum coke powder (Shanxi New Fashion Furnace Industry Group Co., Ltd., Taiyuan, China) was used as the carbon source. Its compositional characteristics were 1.36 wt.% moisture, 0.19 wt.% ash, 13.20 wt.% volatile matter, 84.02 wt.% fixed carbon, and 1.23 wt.% sulfur. The low ash content indicates a relatively small amount of inorganic residue, suggesting that the influence of mineral impurities on SiC formation is likely limited under the present experimental conditions, although the contribution of individual trace metallic species cannot be completely excluded. Silicon powder (Si, ≥99%, Shanghai Aiken Co., Ltd., Shanghai, China) was used as the silicon source. The particle size distribution, measured by laser diffraction, was D10 = 0.652 μm, D50 = 1.704 μm, and D90 = 7.945 μm. The volume-weighted mean diameter is approximately 2.5 μm, and the standard deviation of the distribution is approximately 1.2 μm.

### 2.2. Sample Preparation

The coke powder was heat-treated under an argon atmosphere (purity ≥ 99.9%, flow rate 100 mL/min) at a heating rate of 5 °C/min to 600, 800, 1000, 1200, and 1400 °C, respectively, and soaked for 3 h at each temperature. The heat-treated coke samples were designated as C-600, C-800, C-1000, C-1200, and C-1400, respectively.

Coke powder and silicon powder were weighed at a molar ratio of n(C):n(Si) = 1:1 based on the stoichiometry of the reaction Si + C → SiC, assuming the fixed carbon content of the coke is approximately 84% (as per the supplier’s specifications), and mixed homogeneously by ball milling in a zirconia jar with zirconia balls (ball-to-powder mass ratio = 3:1) at 200 rpm for 4 h. The mixed powders were uniaxially cold-pressed into cylindrical specimens (Φ20 mm × 20 mm) at 25 MPa. The resulting specimens were designated as CS. For the graphite-burial treatment, each compact was placed in a high-purity alumina crucible (Al_2_O_3_ ≥ 99.5%), completely surrounded by graphite powder, and heat-treated in a sealed furnace at 1000, 1100, 1200, 1300, or 1400 °C for 3 h. During heating, at temperatures of 1000 °C and above, the graphite powder reacted with residual oxygen in the sealed furnace to generate CO, creating a reducing atmosphere. Based on the literature [23], the resulting gas composition can be approximated as 35% CO and 65% N_2_; no external gas flow was applied. The CS specimens were heated to 1000, 1100, 1200, 1300, and 1400 °C, respectively, and held for 3 h at each target temperature. The heating rate was controlled at 5 °C/min for temperatures below 1000 °C and 3 °C/min for temperatures above 1000 °C. After heat treatment, the specimens were furnace-cooled to room temperature (cooling rate of approximately 5 °C/min) and collected for phase and microstructural characterization. To ensure reproducibility, three independent specimens were prepared and tested for each temperature, and the reported quantitative data represent the average values with standard deviations.

### 2.3. Characterization

The pyrolysis behavior and mass loss of the coke were analyzed by thermogravimetry and differential scanning calorimetry (TG-DSC, STA 449C, Netzsch, Selb, Germany) at a heating rate of 10 °C/min in an argon atmosphere. The particle size distribution of the coke powder and silicon powder was measured using a laser particle size analyzer (TOPSIZER plus, Omec, Zhuhai, China). The phase compositions of the raw coke, heat-treated coke, and CS products were analyzed by X-ray diffraction (XRD, X’Pert Pro MPD, Philips, Eindhoven, The Netherlands) using Cu Kα radiation. The XRD patterns of the raw and heat-treated coke samples were recorded over a 2θ range of 10–80°, whereas those of the heat-treated CS products were recorded over a 2θ range of 10–90°. The step size was 0.02° and the scan rate was 2°/min. Background subtraction was performed using the HighScore Plus software (version 3.0.4) with a cubic spline baseline correction. It should be noted that the presence of amorphous phases (such as SiO_2_) may contribute to the diffuse scattering background in the XRD patterns, which could affect the accuracy of phase quantification. The phase fractions reported in XRD patterns are therefore semi-quantitative estimates based on the integrated intensities of the crystalline peaks after background subtraction. For each identified crystalline phase i, the relative crystalline phase fraction was estimated according to Fi = Ai/∑Aj × 100%, where Ai represents the summed integrated area of the selected diffraction peaks assigned to phase i, and ∑Aj represents the total integrated area of the selected crystalline diffraction peaks. The resulting values represent normalized semi-quantitative crystalline fractions rather than absolute bulk phase contents, because amorphous and poorly crystalline contributions are not independently quantified. The structural evolution of the coke-derived carbon was evaluated by a laser confocal micro-Raman spectrometer (Raman, inVia Qontor, Renishaw, Wotton-under-Edge, UK) using a 532 nm excitation laser. The spectra were recorded in the range of 500–3000 cm^−1^ with a spectral resolution of ~1 cm^−1^. Background subtraction was performed using a linear baseline correction. The intensity ratio I_D_/I_G_, calculated from the integrated areas of the D and G bands after baseline correction, is denoted here as I_D_/I_G_ following the common convention in carbon Raman spectroscopy. The positions and full widths at half maximum (FWHM) of the D and G bands were also extracted from the fitted spectra using a Lorentzian function for the D band and a Gaussian function for the G band. The integrated area ratio was used rather than the peak height ratio because it is less sensitive to peak broadening effects and provides a more robust measure of the relative abundance of D and G band contributions. The D band was fitted with a Lorentzian function, consistent with its origin from a double-resonance process involving defect-induced lifetime broadening, while the G band was fitted with a Gaussian function to account for inhomogeneous broadening due to the distribution of crystallite sizes and orientations.

The morphologies and microstructures of the raw coke, heat-treated coke, and CS products were examined by field-emission scanning electron microscopy (FESEM, Nova400 NanoSEM, FEI, Hillsboro, OR, USA). High-resolution transmission electron microscopy (HRTEM, JEM-2100UHR, JEOL, Tokyo, Japan) equipped with energy-dispersive X-ray spectroscopy (EDS) was employed to characterize the SiC whiskers formed in the heat-treated CS samples. The TEM was operated at an accelerating voltage of 200 kV. Samples for TEM observation were prepared by dispersing the heat-treated products in ethanol by ultrasonication, followed by drop-casting onto holey carbon-coated copper grids.

## 3. Results and Discussion

### 3.1. Pyrolysis Behavior and Structural Evolution of Coke

**Figure 1:**
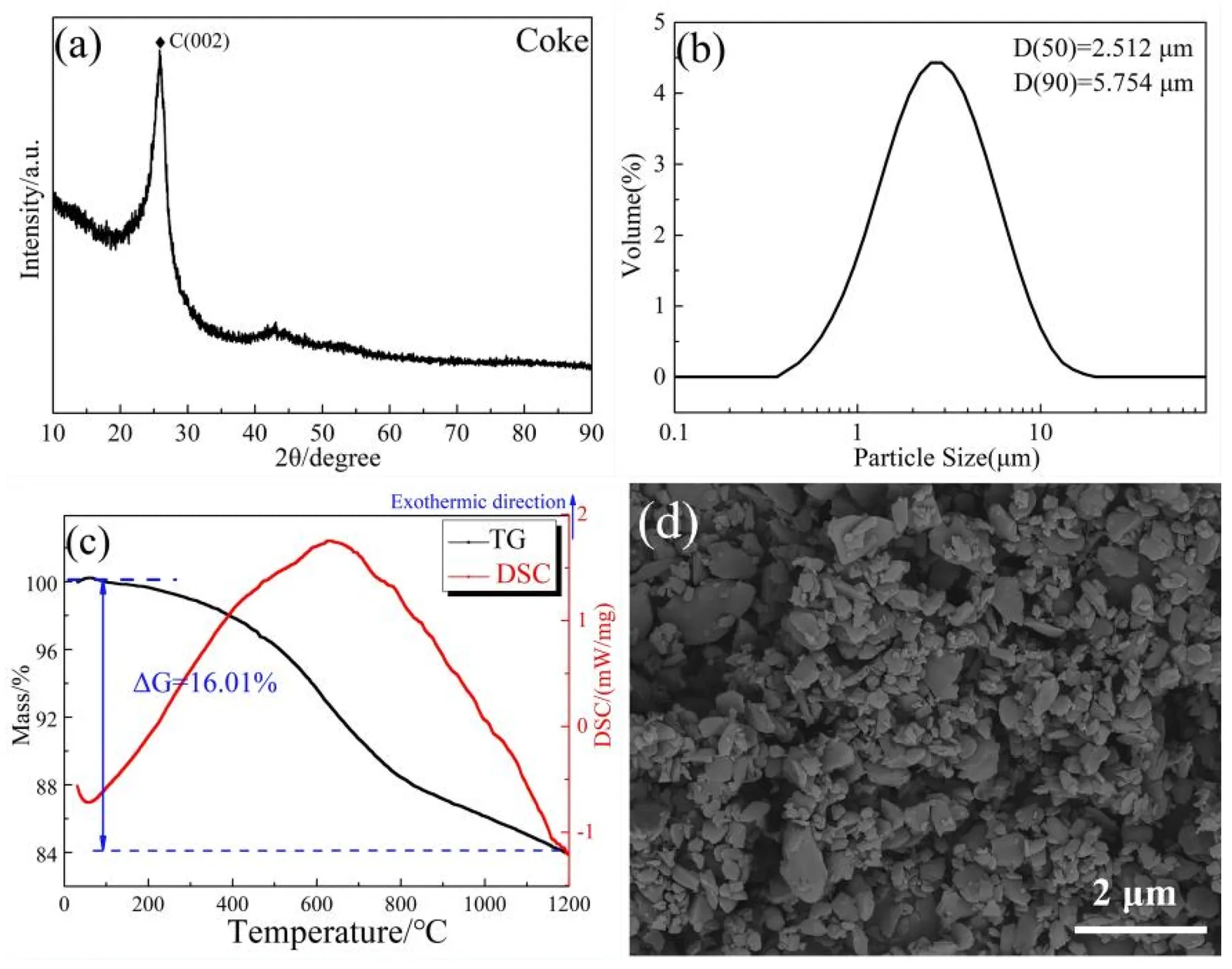
(**a**) XRD pattern, (**b**) particle-size distribution, (**c**) TG-DSC curves, and (**d**) SEM morphology of the raw coke.

The raw coke was characterized using XRD, particle size analysis, TG-DSC, and SEM, with the results summarized in Figure 1.

The XRD pattern (Figure 1a) exhibits a strong diffraction peak at 2θ ≈ 26°, corresponding to the C(002) planes (ICDD 01-089-8487). The peak is relatively broad and asymmetric, which reflects a distribution of interlayer spacings and the presence of disordered stacking of carbon layers. A weak feature near 2θ ≈ 43° is attributed to the in-plane (10) band of the carbon structure. The coke powder presents a narrow unimodal particle-size distribution (Figure 1b), with D50 = 2.512 μm and D90 = 5.754 μm. The TG curve (Figure 1c) shows a total mass loss of 16.01% from room temperature to 1200 °C, which is accompanied by a broad exothermic DSC profile. The SEM image (Figure 1d) reveals irregular plate-like and block-like particles with rough surfaces. Fine particles are attached to the surfaces of larger particles, and irregular interparticle voids are clearly visible.

**Figure 2:**
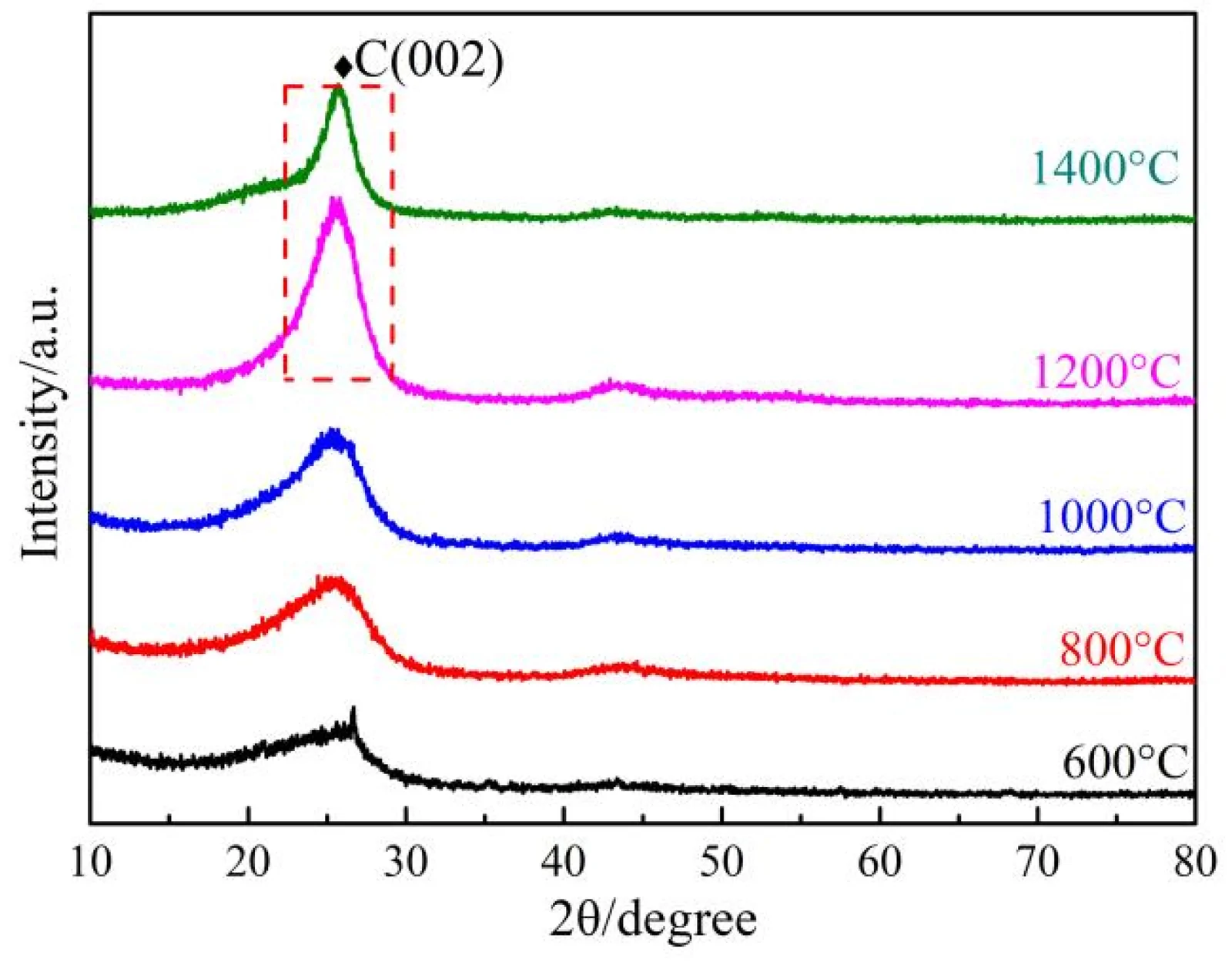
XRD patterns of coke after heat treatment at 600–1400 °C.

To monitor the structural evolution of the coke during heat treatment, the samples treated at different temperatures were examined by XRD. The diffraction patterns are presented in Figure 2.

All heat-treated coke specimens exhibit a diffraction peak corresponding to the C(002) planes near 25–26°, as shown in Figure 2. At 600 °C and 800 °C, the C(002) reflection is broad, indicating a predominantly turbostratic carbon structure. The change is particularly pronounced at 1200 °C and 1400 °C, where the C(002) peak is notably narrowed and heightened. Concurrently, a weak in-plane(10) band reflection near 43° becomes discernible at these elevated temperatures. The progressive sharpening of the C(002) peak reflects the increased stacking order of the carbon layers and the growth of carbon microcrystallites. With increasing temperature, the (002) diffraction peak gradually becomes narrower and more clearly defined. Although the peak height of the 1400 °C sample is slightly lower than that of the 1200 °C sample, absolute diffraction intensity may be influenced by sample packing and preferred orientation, and is therefore not used here as the principal indicator of structural ordering.

To quantitatively evaluate the evolution of the carbon layer stacking structure and crystalline ordering with increasing heat-treatment temperature, the structural parameters associated with the C(002) reflection were further determined. The interlayer spacing d_002_ was calculated using Bragg’s law, and the crystallite stacking height Lc was determined from the Scherrer equation based on the FWHM of the C(002) peak, using Cu Kα radiation (λ = 0.15406 nm) and a shape factor of K = 0.89; the calculated structural parameters are summarized in Table 1. The 2θ_002_ peak positions in Table 1 were determined by curve fitting using the Pearson VII function after background subtraction in the HighScore Plus software, allowing the peak position to be determined with a precision better than the step size (0.02°).

Table 1 shows the crystal structure parameters of coke after heat treatment at 600–1400 °C. As the heat-treatment temperature increases from 600 to 1400 °C, the 2θ_002_ peak generally shifts toward higher diffraction angles, particularly above 1000 °C. Correspondingly, the interlayer spacing d_002_ shows an overall decrease from approximately 0.354 nm at 600–1000 °C to 0.34699 nm at 1400 °C. This decrease indicates progressive contraction of the average spacing between adjacent carbon layers and an improvement in the stacking order of the carbon structure. Meanwhile, the FWHM of the (002) peak decreases markedly from 5.785° at 600 °C to 2.305° at 1400 °C, indicating a gradual sharpening of the diffraction peak with increasing temperature. Such peak narrowing is generally associated with enhanced structural ordering, an increase in coherent crystallite dimensions, and a reduction in structural disorder along the stacking direction. Consistently, the crystallite stacking height Lc increases from 1.39 nm at 600 °C to 3.50 nm at 1400 °C, demonstrating the progressive growth of the coherent stacking domains along the c-axis. Overall, these changes reveal that heat treatment promotes the rearrangement and ordering of the carbon layers. The structural evolution is relatively limited below 1000 °C, whereas a more pronounced decrease in d_002_ and a rapid increase in Lc occur between 1000 and 1400 °C, suggesting accelerated development of short-range graphitic ordering at higher temperatures. Nevertheless, the d_002_ value at 1400 °C remains substantially larger than that of ideal graphite (0.3354 nm), while the Lc value remains relatively small. Therefore, the coke retains a predominantly turbostratic carbon structure, although its degree of structural ordering is significantly enhanced with increasing heat-treatment temperature [24].

**Figure 3:**
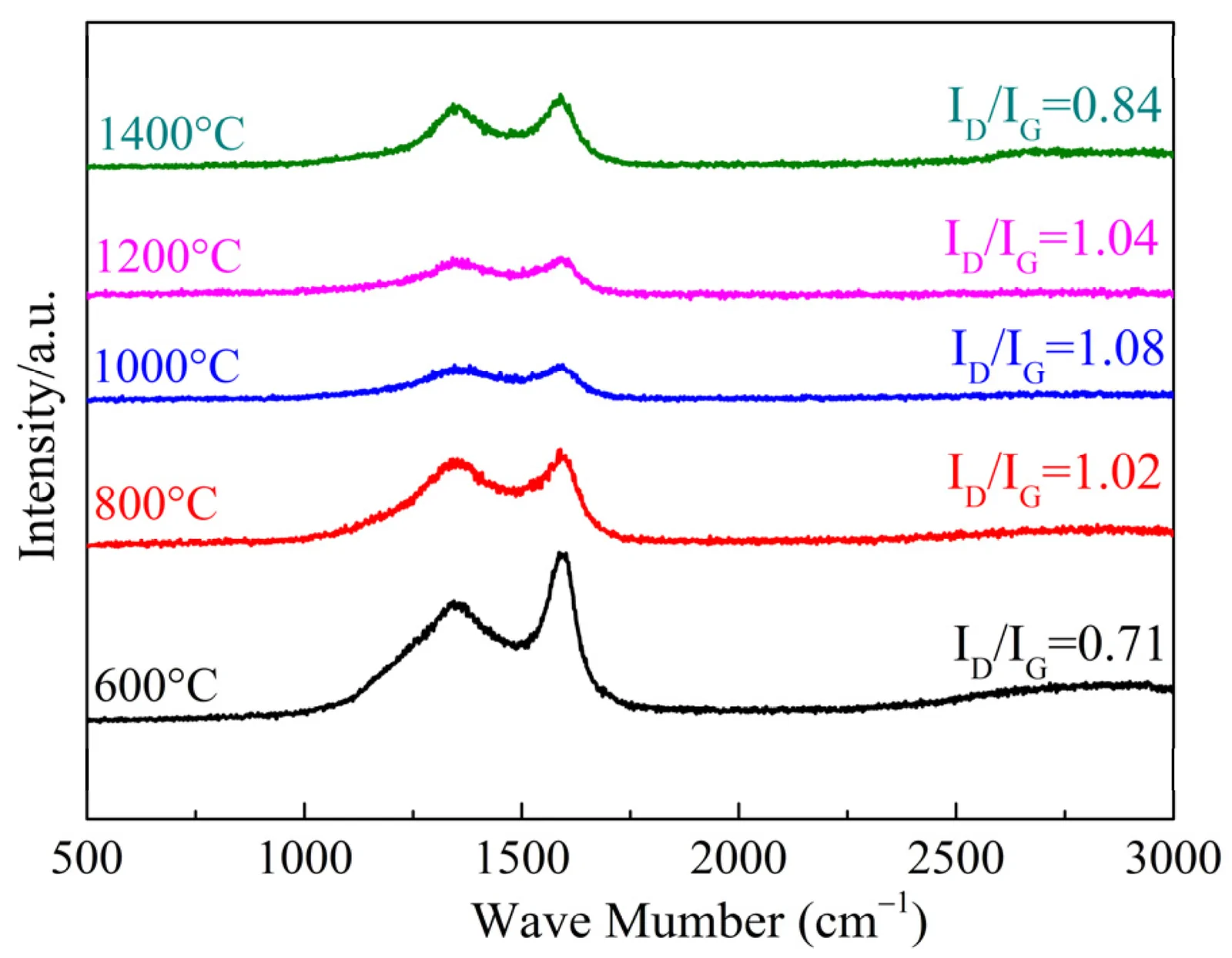
Raman spectra of coke after heat treatment at 600–1400 °C.

To further assess the microstructural ordering of the coke during heat treatment, Raman spectroscopy was performed on the thermally treated samples, and the spectra are presented in Figure 3. The Raman spectra of the coke-derived carbon exhibit two characteristic peaks at 1344 cm^−1^ and 1604 cm^−1^, which are attributed to the D and G bands, respectively [25]. The D band arises from a double-resonance Raman process and is associated with structural defects, vacancies, and disordered domains, whereas the G band corresponds to the in-plane stretching vibration of sp^2^-hybridized carbon atoms within the graphite-like microcrystallites [26]. The intensity ratio of the D band to the G band, denoted as R (R = I_D_/I_G_), was employed to monitor the structural ordering of the coke [27]. However, it should be noted that the relationship between I_D_/I_G_ and structural disorder is not strictly monotonic across all stages of carbon structural evolution. In the present study, the observed variation in I_D_/I_G_ with temperature is interpreted qualitatively as an indication of changes in the degree of structural ordering.

The Raman spectra in Figure 3 exhibit the characteristic D-band and G-band features of the carbonaceous material. The detailed Raman spectral parameters are summarized in Table 2. The I_D_/I_G_ ratio changes non-monotonically with increasing temperature: 0.71 at 600 °C, 1.02 at 800 °C, 1.08 at 1000 °C, 1.04 at 1200 °C, and 0.84 at 1400 °C. The fitted Raman parameters are summarized in Table 2. From 600 to 1000 °C, the I_D_/I_G_ ratio increases from 0.71 to 1.08, accompanied by an increase in the FWHM of both the D band (169.57 to 182.85 cm^−1^) and the G band (77.19 to 102.66 cm^−1^). This evolution is consistent with structural rearrangement of the coke-derived carbon during devolatilization and the development of relatively small aromatic domains with heterogeneous local environments. Above 1000 °C, a different trend becomes evident. The D-band FWHM decreases markedly to 117.98 cm^−1^ at 1400 °C, while the I_D_/I_G_ ratio decreases to 0.84. The G band also becomes narrower relative to that at 1000 °C and shifts to 1589.32 cm^−1^ at 1400 °C. These changes suggest a reduction in structural heterogeneity and enhanced short-range ordering of the sp^2^-bonded carbon network. This interpretation is further supported by the XRD-derived structural parameters. With increasing heat-treatment temperature, d_002_ decreases, the C(002) FWHM narrows, and Lc increases, indicating contraction of the average interlayer spacing and growth of the coherent stacking domains. Therefore, the combined Raman and XRD results indicate that the coke undergoes progressive structural rearrangement and enhanced short-range ordering at elevated temperatures, particularly above 1000 °C, while retaining a predominantly turbostratic carbon structure.

**Figure 4:**
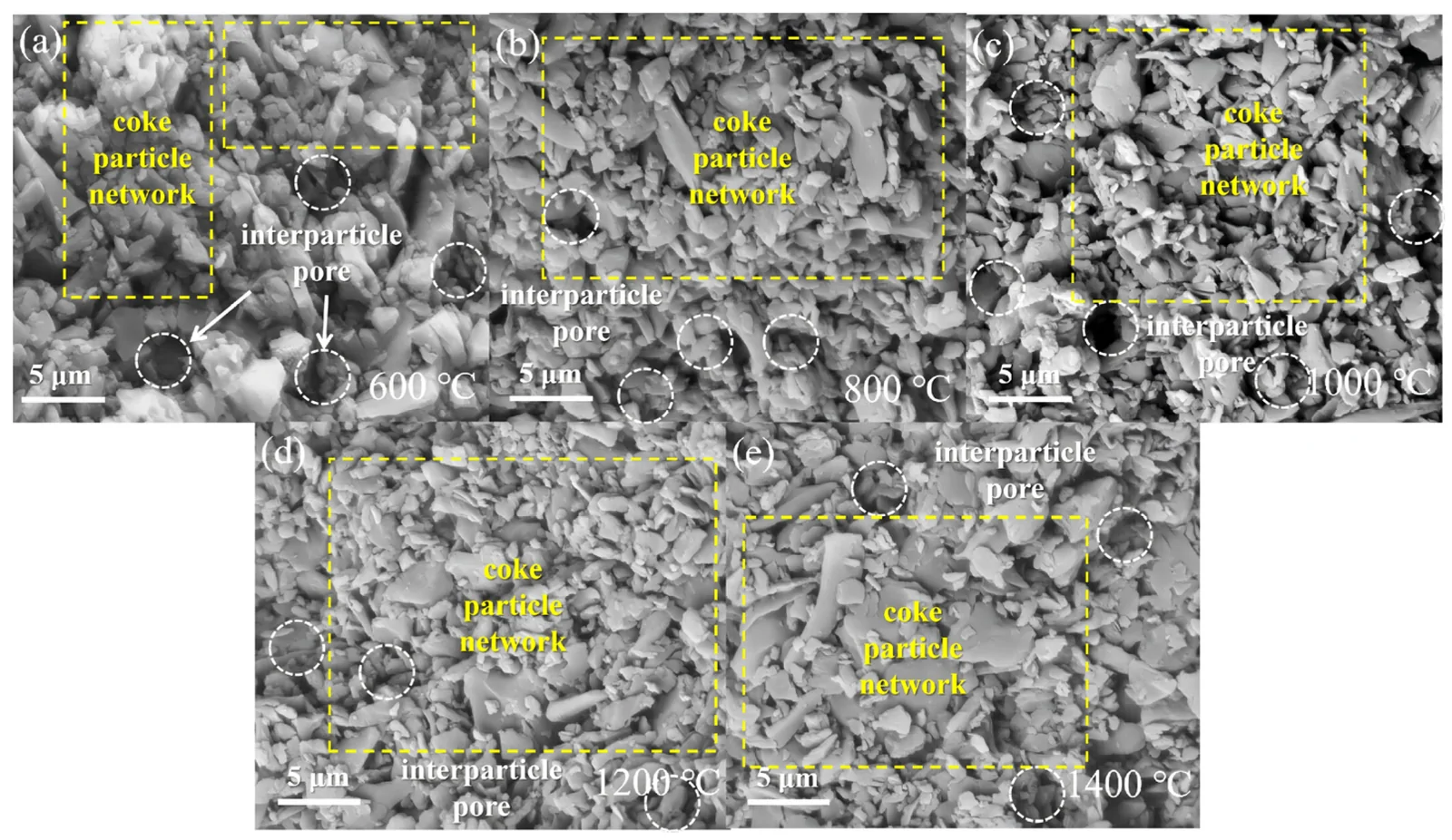
SEM images of the coke after heat treatment at 600–1400 °C. (**a**) 600 °C, (**b**) 800 °C, (**c**) 1000 °C, (**d**) 1200 °C, and (**e**) 1400 °C.

The morphological stability of the coke particles after heat treatment was examined via SEM, and the micrographs are presented in Figure 4.

The SEM images in Figure 4 reveal that the coke particles maintain an irregular plate-like and block-like morphology with rough surfaces and fragmented edges across all heat treatment temperatures from 600 °C to 1400 °C. As highlighted by the yellow dashed boxes, the coke particles remain interconnected and form a continuous particle network throughout the investigated temperature range. Representative interparticle pores, indicated by the white dashed circles and arrows, are also retained between adjacent particles. Despite the internal microstructural ordering indicated by the XRD and Raman results, no obvious morphological evolution, such as particle agglomeration or extensive fusion, is observable. The persistence of both the interconnected particle network and interparticle pores indicates that the overall coke framework remains morphologically stable during heat treatment. The interparticle voids and the fragmented features remain consistent with the raw powder characterization, confirming the overall morphological stability of the coke framework during thermal processing.

The structural evolution of the coke from a defect-rich turbostratic structure to a more structurally ordered turbostratic state (as evidenced by Figure 2 and Figure 3) may influence the nucleation and growth behavior of SiC whiskers by altering the density of active sites and the stability of the carbon framework during the synthesis reaction. The preservation of this interconnected particle network and porous structure (Figure 4) at elevated temperatures is an important morphological factor for its role as a carbon source in subsequent SiC synthesis. Meanwhile, the stable carbon framework provides reaction interfaces and a sustained carbon source for SiC formation at higher temperatures.

### 3.2. Phase Evolution of CS

**Figure 5:**
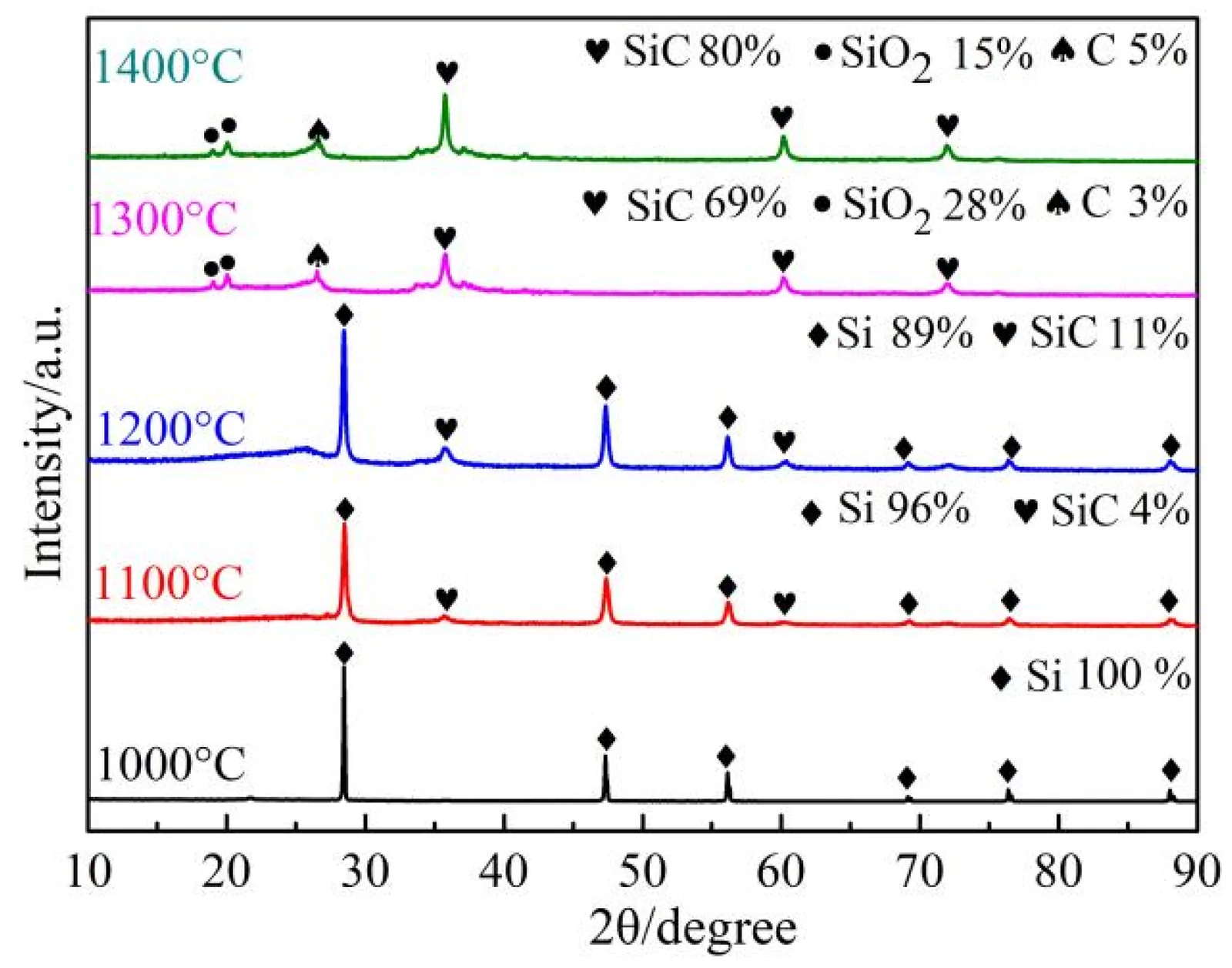
XRD patterns and semi-quantitative crystalline phase fractions of CS samples after heat treatment at 1000–1400 °C.

The XRD patterns and semi-quantitative crystalline phase fractions of CS samples after heat treatment at 1000–1400 °C are shown in Figure 5. The reported percentages correspond to the normalized fractions of the identified crystalline phases. Amorphous components and poorly crystalline phases are not quantitatively included. These values do not represent the total phase composition of the samples because amorphous components and poorly crystalline carbon are not independently quantified in the present analysis.

As shown in Figure 5, the XRD patterns of the specimens treated at 1000 °C exhibit exclusively sharp diffraction peaks corresponding to elemental Si, indicating that no discernible reaction occurred at this temperature. At 1100 °C, the identified crystalline phases consisted predominantly of Si, with a small fraction of crystalline SiC. At 1200 °C, the relative crystalline SiC fraction increased, indicating enhanced SiC formation with increasing temperature. At 1300 °C, the detected crystalline phases were mainly composed of SiC, together with crystalline SiO_2_, and a minor carbon contribution. The crystalline Si peaks disappear completely, and SiC becomes the dominant phase, accounting for 69% of the identified crystalline phase fraction, accompanied by 28% crystalline SiO_2_ and 3% crystalline carbon contribution. The crystalline SiO_2_ detected at 1300 °C may originate, at least in part, from the native oxide layer on the surface of the Si powder. During heat treatment and subsequent cooling, Si–O-containing species derived from this surface oxide may undergo oxidation and/or deposition to form SiO_2_. It should be noted that the reported SiO_2_ fraction is a normalized semi-quantitative fraction of the identified crystalline phases rather than an absolute bulk mass fraction of SiO_2_ in the specimen. As the temperature further increases to 1400 °C, the SiC content continues to rise to 80%, while the SiO_2_ content decreases to 15%, the concurrent decrease in SiO_2_ and increase in SiC at 1400 °C indicates that elevated temperatures further promote SiC formation. For samples treated below 1100 °C, the carbon fraction was not quantitatively assigned because the coke-derived carbon retained a broad and weak diffraction feature, making reliable separation and quantification of carbon from the other phases difficult using the present XRD method. Overall, under the coke-burial conditions, the reaction between coke and Si yields only a limited amount of SiC at 1100 °C and 1200 °C. At 1300 °C, the Si peaks vanish completely, indicating that crystalline elemental Si is no longer detectable by XRD, and the SiC fraction continues to increase as the temperature rises to 1400 °C.

### 3.3. Microstructural Evolution of CS

**Figure 6:**
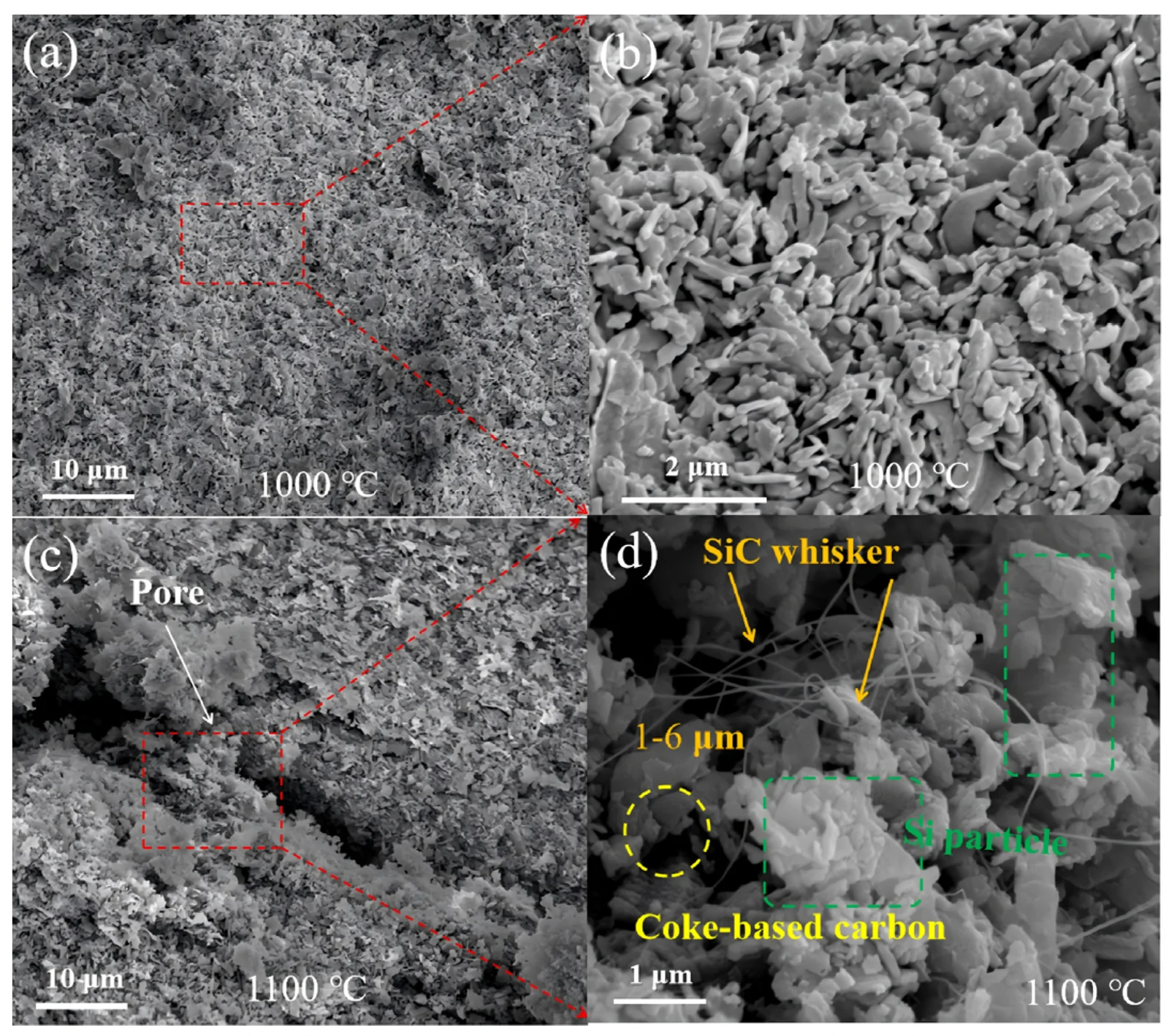
SEM images of CS after heat treatment at 1000–1100 °C. (**a**,**b**) 1000 °C, and (**c**,**d**) 1100 °C. The enlarged SEM images in (**b**) and (**d**) correspond to the selected regions in (**a**) and (**c**), respectively.

The early-stage microstructure of the CS compacts heat-treated at 1000 °C and 1100 °C was observed via SEM, and the micrographs are presented in Figure 6.

As shown in Figure 6, the specimen heat-treated at 1000 °C (Figure 6a,b) exhibits a densely packed granular microstructure consisting of unreacted coke and Si particles, with no observable one-dimensional products. When the temperature increases to 1100 °C (Figure 6c,d), a limited number of slender SiC whiskers begin to form, mainly within the pore regions and around the Si-containing particles. To more clearly reveal the morphology of these early-stage whiskers, a higher-magnification SEM image is provided in Figure 6d. The SiC whiskers, residual Si particles, and coke-derived carbon are directly identified in the image. Based on the whiskers visible in this high-magnification field, their representative lengths are approximately 1–6 μm. The preferential occurrence of these whiskers within the pore regions and near Si particles suggests that the pores provide favorable local regions for the initial formation and growth of SiC whiskers.

**Figure 7:**
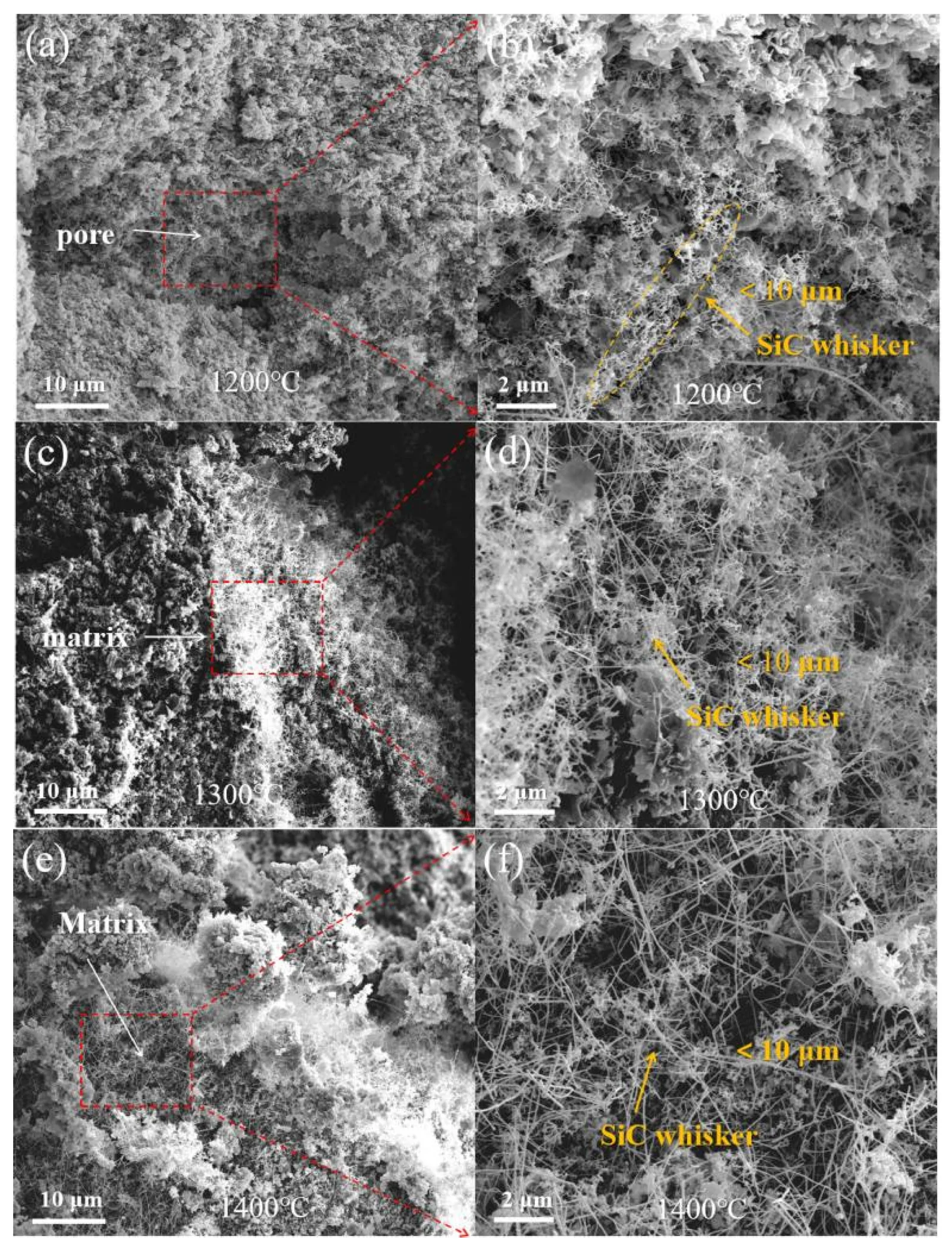
SEM images of CS after heat treatment at 1200–1400 °C. (**a**,**b**) 1200 °C, (**c**,**d**) 1300 °C, and (**e**,**f**) 1400 °C.

To further elucidate the morphological evolution of the reaction products at elevated temperatures, the fractured surfaces of the CS compacts heat-treated at 1200 °C, 1300 °C, and 1400 °C were examined, and the micrographs are shown in Figure 7.

As shown in Figure 7, the location and quantity of the SiC whiskers exhibit a pronounced temperature-dependent evolution. At 1200 °C (Figure 7a,b), SiC whiskers are clearly observed within the pore regions of the coke-derived matrix. Compared with the 1100 °C sample, both the number and spatial extent of the whiskers increase, while their growth remains predominantly localized within the interconnected pore channels. The whiskers visible in the high-magnification image are generally below approximately 10 μm in length. This localized distribution indicates that the pore structure provides favorable transport pathways and reaction sites for the early-stage formation of SiC whiskers. When the temperature increases to 1300 °C (Figure 7c,d), a pronounced increase in the amount of SiC whiskers is observed. The whiskers are no longer primarily confined to isolated pore regions but extend over large areas of the matrix surface, forming a much denser interconnected network. The simultaneous increase in whisker abundance and expansion of the growth region indicates that SiC formation is significantly accelerated at 1300 °C, which may be associated with enhanced reaction kinetics and mass transport of reactive Si-containing and C-containing species. When the temperature rises to 1400 °C (Figure 7e,f), the SiC whiskers form an extensively interconnected network that covers a large fraction of the matrix surface. Compared with the 1300 °C sample, the whisker network becomes denser and more uniformly distributed. Based on the representative SEM observations in Figure 7f, the lengths of the visible SiC whiskers are below approximately 10 μm. Overall, the SEM observations reveal a progressive evolution from localized pore-confined whisker formation at 1200 °C to widespread matrix-surface coverage at 1300 °C and finally to a dense interconnected whisker network at 1400 °C. This temperature-dependent morphological evolution demonstrates that increasing temperature plays a crucial role in determining the morphology and spatial distribution of SiC whiskers [28].

**Figure 8:**
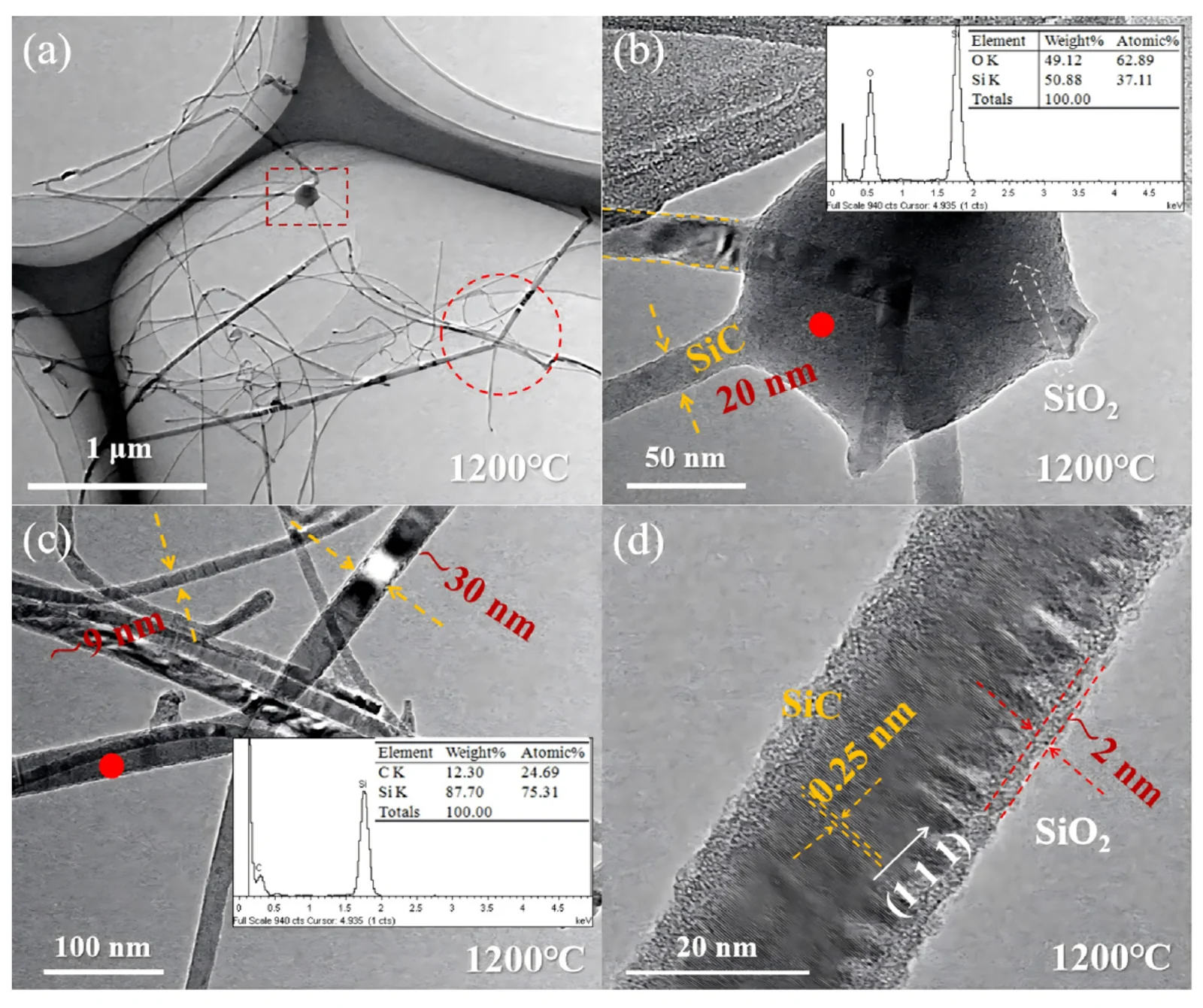
TEM, HRTEM and EDS analyses of SiC whiskers formed at 1200 °C. (**a**) TEM image, (**b**) HRTEM image with the corresponding EDS spectrum of the shell region, (**c**) HRTEM image with the EDS spectrum of the core region, and (**d**) high-magnification HRTEM image of the SiC whiskers formed at 1200 °C.

It should be noted that the TEM characterization of the product formed at 1100 °C was not feasible because the extremely limited quantity of whiskers at this temperature was insufficient for statistical sampling or reliable observation. Therefore, the 1200 °C specimen was selected to represent the early stage of whisker development. To further confirm the crystallographic structure and elemental composition of the products formed at 1200 °C, TEM, HRTEM, and EDS analyses were conducted on the CS sample, and the results are presented in Figure 8.

As displayed in Figure 8a, the whiskers formed at 1200 °C exhibit a high aspect ratio, with representative diameters of approximately 9–30 nm in the observed TEM fields. The EDS analysis of the outer layer in Figure 8b indicates a high content of Si and O, confirming the presence of an amorphous SiO_2_ layer surrounding the whisker. In contrast, the EDS spectrum of the inner region (inset of Figure 8c) primarily contains Si and C, identifying the inner core as SiC. The HRTEM image in Figure 8d displays continuous lattice fringes with an interplanar spacing of approximately 0.25 nm, which corresponds to the (111) plane of β-SiC [29]. Meanwhile, an amorphous surface layer with a local thickness of approximately 2 nm is visible in the HRTEM image and is assigned to SiO_2_ on the basis of the EDS result. The observed surface SiO_2_ layer indicates that oxidation/deposition of Si–O-containing species may have occurred during synthesis and/or subsequent cooling and specimen exposure.

**Figure 9:**
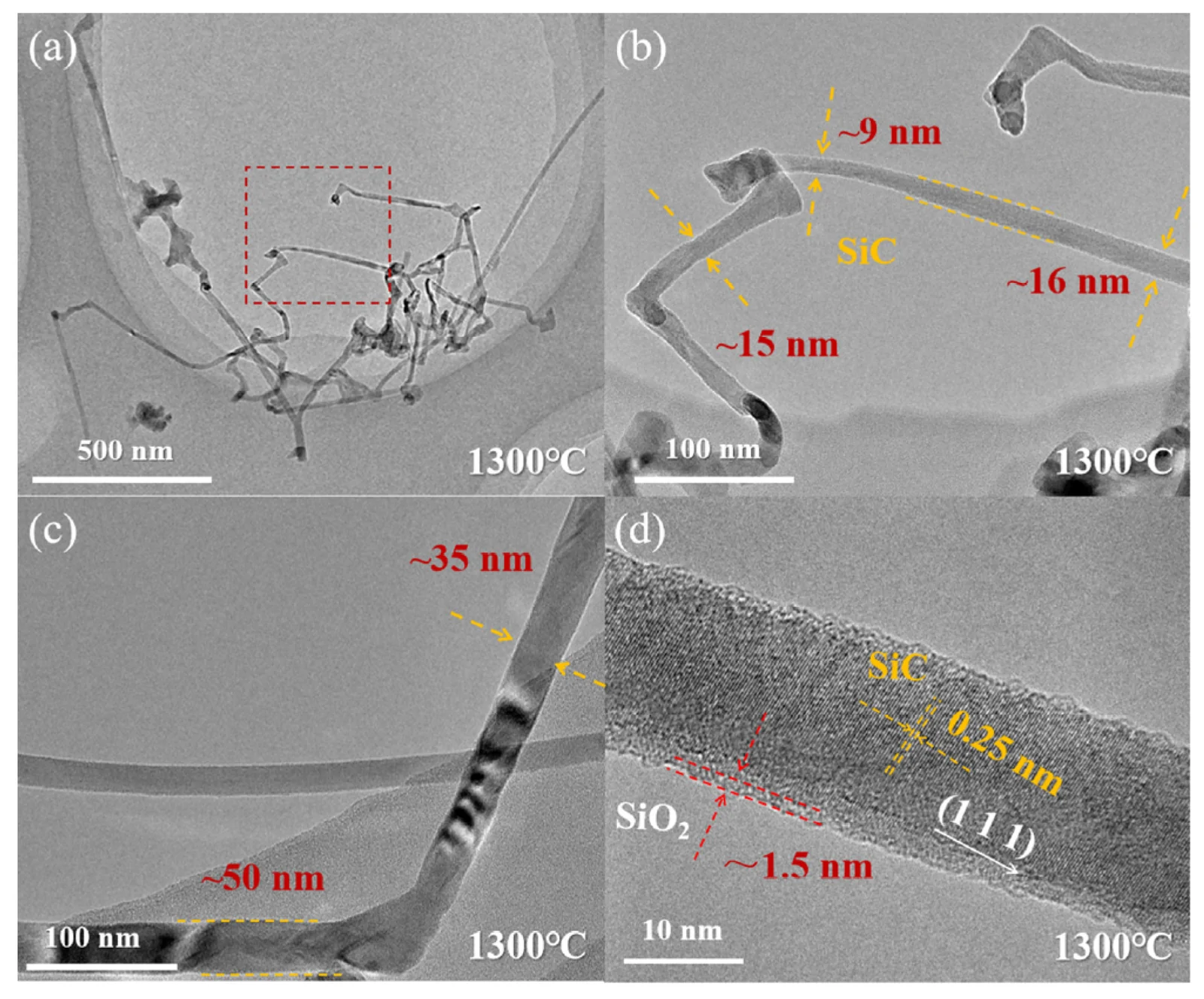
TEM and HRTEM images of SiC whiskers formed at 1300 °C. (**a**) low-magnification TEM image; (**b**) enlarged TEM image of the region marked in (**a**); (**c**) medium-magnification TEM images; (**d**) HRTEM image.

Because 1300 °C corresponds to the temperature at which the crystalline Si reflections disappear and the SEM morphology changes from localized to widespread whisker formation, additional TEM/HRTEM characterization was performed specifically on the 1300 °C specimen (Figure 9).

As shown in Figure 9a–c, the SiC whiskers formed at 1300 °C exhibit well-developed one-dimensional morphologies. Representative whisker diameters observed in the present TEM images range from approximately 9 to 50 nm, showing a broader diameter distribution and more pronounced radial development than those observed at 1200 °C. The HRTEM image in Figure 9d exhibits lattice fringes with an interplanar spacing of approximately 0.25 nm, consistent with the (111) planes of β-SiC. An amorphous SiO_2_-rich outer layer is clearly observed on the surface of the SiC whisker, and its local thickness is approximately 1.5 nm, as directly indicated in Figure 9d. No terminal droplets are evident in the TEM fields examined; therefore, the present TEM observations do not provide direct evidence for a classical liquid-mediated VLS growth process. Therefore, the abrupt increase in whisker abundance at 1300 °C is interpreted as evidence of strongly accelerated SiC formation rather than proof of a transition to a VLS mechanism.

**Figure 10:**
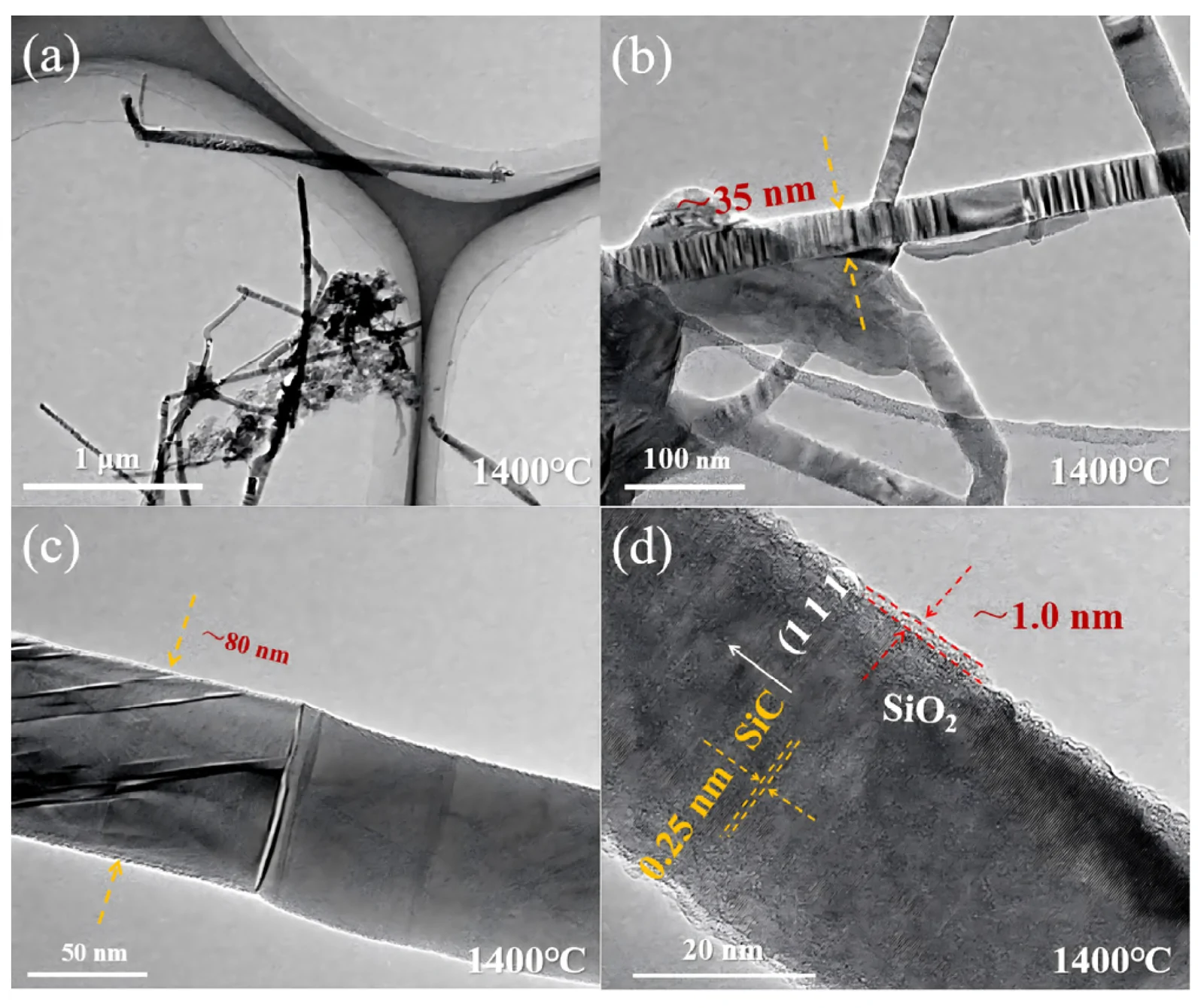
TEM and HRTEM images of SiC whiskers formed at 1400 °C. (**a**) elongated morphology of the whiskers; (**b**,**c**) high-density stacking faults with bamboo-like segmented structures; (**d**) high-resolution lattice fringes with a 0.25 nm interplanar spacing.

To investigate the morphological and crystallographic characteristics of the well-developed whiskers formed at higher temperatures, TEM and HRTEM analyses were performed on the 1400 °C CS sample, and the results are presented in Figure 10.

As shown in Figure 10, the SiC whiskers formed at 1400 °C exhibit a well-developed one-dimensional morphology with high aspect ratios (Figure 10a). Compared to the whiskers observed at 1200 °C, the diameters are notably larger, with representative diameters of approximately 35–80 nm in the TEM images shown in Figure 10b,c. A prominent feature of these whiskers is the presence of high-density periodic contrast bands running perpendicular to the growth axis. These parallel bands are characteristic of stacking faults, which are commonly generated during the rapid growth of cubic SiC along the <111> direction [30]. The distinct bamboo-like segmented morphology visible in the thicker whisker (Figure 10c) is a morphological manifestation of these extensive planar defects. The HRTEM image in Figure 10d reveals continuous lattice fringes with an interplanar spacing of 0.25 nm, corresponding to the (111) planes of β-SiC. An amorphous SiO_2_-rich outer layer is also observed at the surface of the SiC whisker. Its local thickness is approximately 1.0 nm, as directly marked in Figure 10d. Compared with the thicker surface layer observed in the lower-temperature samples, the locally observed amorphous outer layer becomes thinner at 1400 °C.

To quantitatively evaluate the temperature-dependent morphological evolution of the SiC whiskers, their average length, diameter, aspect ratio, and apparent areal density were determined from SEM and TEM observations, as summarized in Table 3. The average whisker length increases from 2.5 ± 0.6 μm at 1100 °C to 3.4 ± 0.7 μm at 1200 °C and 4.4 ± 0.9 μm at 1300 °C, before remaining at a comparable level of 4.0 ± 0.8 μm at 1400 °C. In contrast, the average diameter increases continuously from 15 ± 5.5 nm at 1100 °C to 19.7 ± 8.5 nm at 1200 °C and 25.0 ± 17.0 nm at 1300 °C, followed by a marked increase to 57.5 ± 31.8 nm at 1400 °C. Accordingly, the aspect ratio increases from approximately 140 ± 40 at 1100 °C to a maximum of 173 ± 80 at 1200 °C, and then decreases to 164 ± 110 at 1300 °C and 70 ± 43 at 1400 °C. These results indicate predominantly axial growth at 1100–1200 °C, followed by simultaneous axial and radial growth at 1300 °C. At 1400 °C, the nearly unchanged whisker length together with the pronounced increase in diameter and decrease in aspect ratio indicates increasingly significant radial coarsening. The apparent areal density also increases from 0.10 ± 0.03 μm^−2^ at 1100 °C to 1.10 ± 0.20 μm^−2^ at 1400 °C, consistent with the progressive densification of the whisker network observed by SEM. Overall, these quantitative trends are consistent with the SEM and TEM observations and support the temperature-dependent evolution of SiC whisker growth.

### 3.4. Growth Mechanism of SiC Whiskers

**Figure 11:**
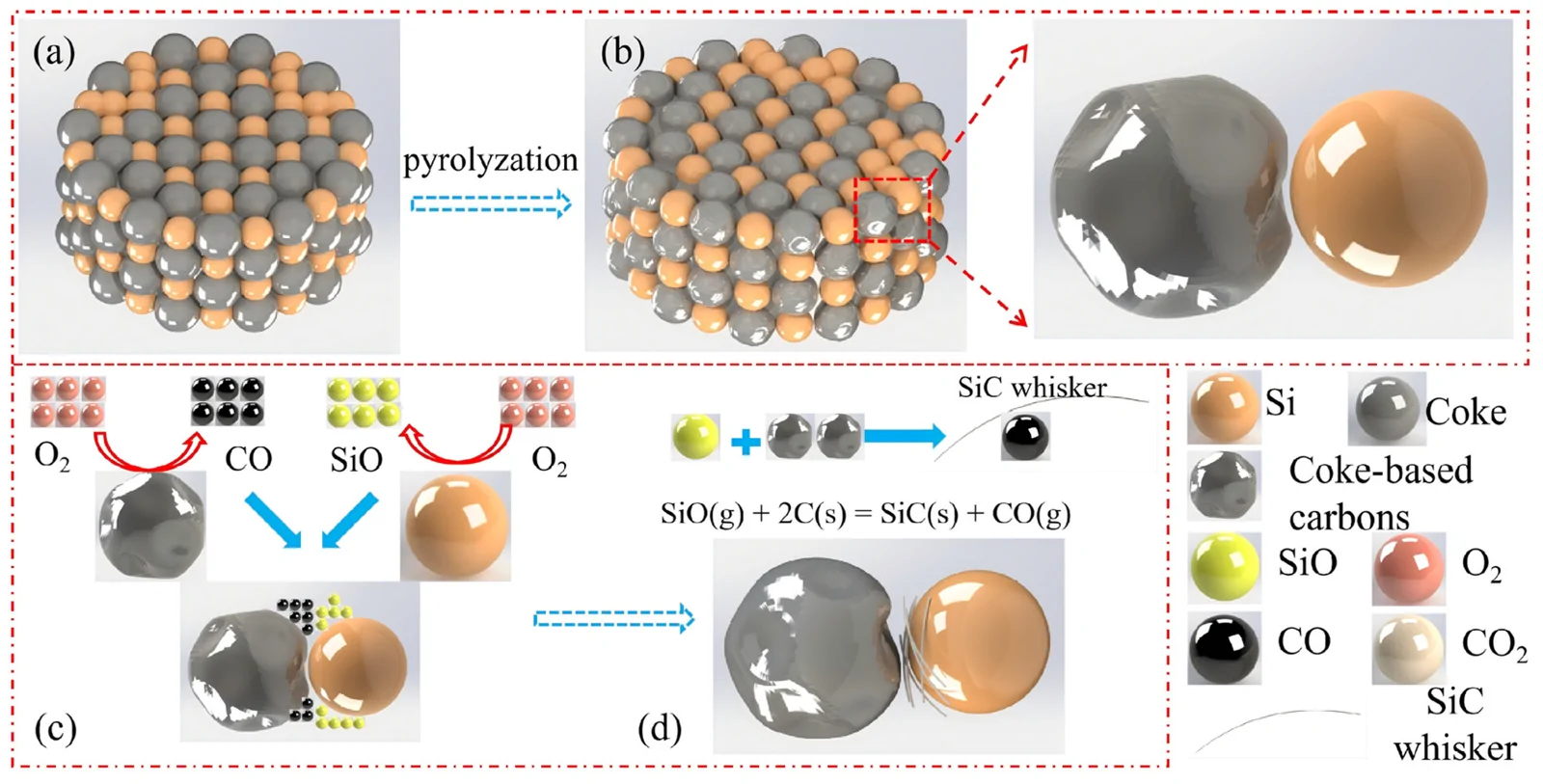
Schematic illustration of the formation mechanism of SiC whiskers from coke and Si powders. (**a**) homogeneous mixing of coke and Si; (**b**) dense packing of particles after pyrolysis; (**c**) vapor–solid (VS) proposed gas-phase-assisted VS-type growth pathway at the interface; (**d**) growth of SiC whiskers at the contact between coke and Si particles.

Based on the phase evolution and microstructural observations discussed above, a schematic illustration of the temperature-dependent formation mechanism of SiC whiskers from coke and Si powders is proposed in Figure 11.

At 1000 °C, the coke-derived carbon and Si particles remain closely packed, and no obvious one-dimensional SiC products are observed. The reaction between Si and carbon is therefore still limited at this stage. When the temperature increases to 1100 °C, a small number of SiC whiskers begin to appear preferentially in the pore regions and around Si-containing particles. The preferential formation of whiskers in these confined pore regions suggests that the interconnected pores provide favorable pathways for the generation, transport, and reaction of gaseous species. Under the graphite-burial conditions, gaseous SiO may be generated through reactions involving Si and oxygen-containing species [31]. Considering the presence of surface oxide species in the Si-containing system, possible SiO-generating reactions include Reactions (1) and (2).

(1) $${2Si(s)+O_{2}(g)=2SiO(g)\;\Delta G}_{1}^{\theta }=718200-263.4T\;(\text{J/mol})$$

(2) $${\mathrm{Si}(s)+\mathrm{CO}(g)=\mathrm{SiO}(g)+C(s)\;\Delta G}_{2}^{\theta }=310000-128.5T\;(\text{J/mol})$$

The generated SiO can subsequently react with coke-derived carbon to form SiC according to the following gas–solid reaction (3):

(3) $${\mathrm{SiO}(g)+2C(s)=\mathrm{SiC}(s)+\mathrm{CO}(g)\;\Delta G}_{3}^{\theta }=-82376+3.76T\;(\text{J/mol})$$

At 1000 °C: ΔG°_3_ ≈ −77.6 kJ/mol; at 1100 °C: ΔG°_3_ ≈ −77.2 kJ/mol; at 1200 °C: ΔG°_3_ ≈ −76.9 kJ/mol; at 1300 °C: ΔG°_3_ ≈ −76.5 kJ/mol; and at 1400 °C: ΔG°_3_ ≈ −76.1 kJ/mol. These negative values confirm that Reaction (3) is thermodynamically favorable throughout the investigated temperature range.

In addition, the formation of SiO_2_-rich surface layers observed by TEM suggests that local reactions involving SiO and CO may occur simultaneously during whisker growth. Therefore, the initial formation of SiC whiskers at 1100 °C is consistent with a gas-phase-assisted vapor–solid (VS) growth process [32]. However, because the gaseous intermediates were not directly detected, this reaction pathway should be regarded as a mechanistic interpretation rather than direct experimental proof.

At 1200 °C, the number of SiC whiskers increases and the whiskers remain preferentially distributed within the pore channels. The increase in temperature is expected to accelerate reaction kinetics and mass transport, thereby increasing the availability and transport rate of reactive Si-containing and C-containing species within the interconnected pores. The persistent porous structure of the coke-derived carbon may provide transport channels and reaction interfaces that are favorable for this proposed gas-phase-assisted growth process. Consequently, SiC whisker formation becomes more pronounced at 1200 °C while retaining the characteristic pore-confined distribution observed at the early growth stage.

A pronounced change in reaction behavior occurs when the temperature increases to 1300 °C. The XRD results show that the diffraction peaks of crystalline Si disappear, while SiC becomes the dominant crystalline phase. Simultaneously, SEM observations reveal that the SiC whiskers are no longer mainly confined to isolated pore regions but become widely distributed over the matrix surface. These changes indicate a substantial acceleration of the conversion of crystalline Si-containing reactants and overall SiC formation and whisker growth between 1200 and 1300 °C. The enhanced reaction behavior may be attributed to the combined effects of accelerated chemical kinetics, increased generation and transport of reactive Si-containing species, and intensified reactions at the Si-containing phase/carbon interfaces [33]. Accordingly, gas-phase-assisted growth may coexist with increasingly important interfacial reactions at this stage, resulting in the rapid formation of a much denser SiC whisker network.

When the temperature further increases to 1400 °C, the matrix surface becomes covered by a dense and interconnected network of SiC whiskers, indicating further enhancement of SiC formation. The TEM observations show pronounced radial coarsening of the whiskers, together with a high density of stacking faults perpendicular to the growth direction. These structural features suggest that the higher reaction rate and enhanced mass transport at 1400 °C may promote rapid incorporation of Si- and C-containing species into the growing β-SiC crystals. As a result, the high-temperature growth stage is characterized not only by an increased population of SiC whiskers but also by substantial radial crystal growth and the development of abundant planar defects.

Overall, the formation of SiC whiskers proceeds through a temperature-dependent multistage process. At 1100–1200 °C, the interconnected pore channels of the coke-derived carbon provide favorable conditions for gas-phase transport, and the observed morphology is consistent with a VS-type SiC nucleation and growth pathway. With increasing temperature to 1300 °C, accelerated reaction kinetics and mass transport substantially enhance SiC formation and extend SiC formation from localized pore regions to the matrix surface. At 1400 °C, further enhancement of mass transfer and crystal-growth kinetics promotes extensive SiC formation, radial coarsening, and the development of stacking faults. Throughout this process, the preserved porous coke framework serves simultaneously as a carbon source, a reaction interface, and a mass-transport network for SiC whisker formation.

**Figure 12:**
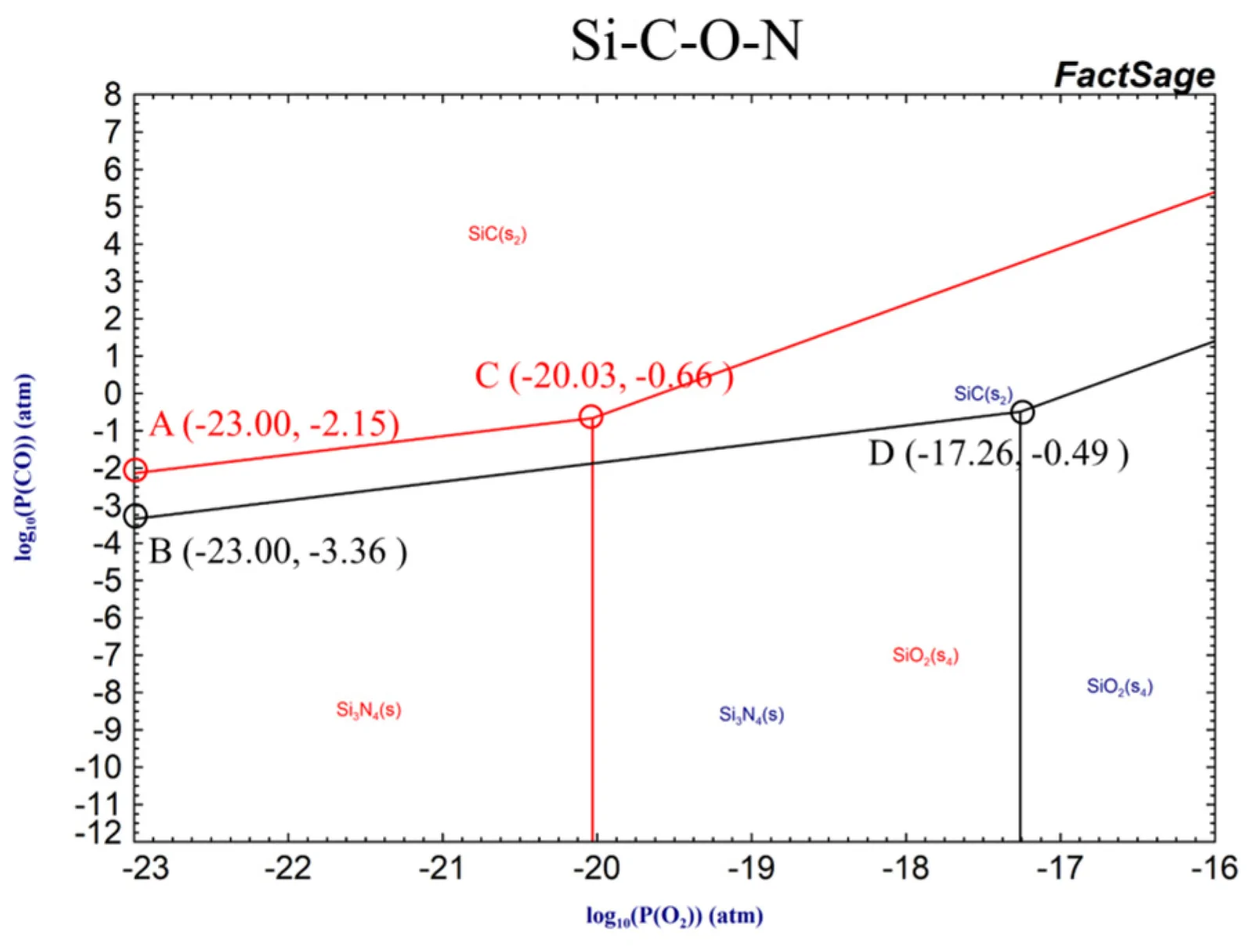
Predominance diagrams of the system Si–C O–N at 1200 °C (red line and text) and 1400 °C (black line and text) calculated by thermodynamic software Factsage^®^.

In this work, the predominance diagrams of the system Si–C–O–N at various temperatures are simulated by the thermodynamic software Factsage^®^ (version 6.2), as shown in Figure 12. It should be noted that these diagrams are intended as a theoretical reference only, illustrating the stability region of SiC with respect to CO and O_2_ partial pressures, rather than a direct simulation of the actual experimental gas composition. The impact of gas (CO and O_2_) partial pressures was analyzed. It can be seen from the figure that SiC can be formed at both 1200 °C and 1400 °C, which lays a theoretical foundation for SiC synthesis. It is worth mentioning that increasing temperature can reduce the partial pressure of CO for SiC formation. For instance, when the P(O_2_) is 10^−23^ atm, with increasing temperature from 1200 °C to 1400 °C, the P(CO) decreases from 10^−2.15^ atm to 10^−3.36^ atm, which means that SiC is produced more easily at a higher temperature. This shift indicates a temperature-dependent expansion of the calculated thermodynamic stability range of SiC under the assumed equilibrium conditions. The thermodynamic results therefore support the feasibility of SiC formation at elevated temperatures.

## 4. Conclusions

In this study, SiC whiskers were synthesized from low-cost coke and silicon powder through a graphite-burial heat-treatment process, and the temperature-dependent structural evolution of coke, phase transformation, microstructural development, and growth mechanisms of the SiC whiskers were systematically investigated.

(1) The raw coke possesses a turbostratic structure with a TG-DSC mass loss of 16.01% and fine particle size (D50 = 2.512 μm). During heat treatment, it undergoes progressive structural rearrangement, growth of coherent stacking domains, and enhanced short-range ordering at 1200–1400 °C while retaining a predominantly turbostratic structure.
(2) SiC whisker formation is strongly temperature-dependent. At 1100–1200 °C, only limited whisker formation occurs and the whiskers are preferentially distributed in pore regions, a morphology consistent with a gas-phase-assisted VS-type growth pathway. At 1300 °C, the crystalline Si reflections disappear and the amount of SiC whiskers increases markedly, with whiskers becoming widely distributed over the matrix surface. No terminal droplets were evident in the examined 1300 °C TEM fields, and therefore the pronounced enhancement of whisker formation at 1300 °C should not be regarded as evidence for a classical VLS mechanism. The enhanced radial growth and densification of the whisker network at 1400 °C are associated with accelerated reaction and mass transport at elevated temperatures.
(3) The synthesized whiskers are crystalline β-SiC with well-developed (111) lattice fringes. The whiskers formed at higher temperatures (1400 °C) exhibit increased diameters (up to 80 nm), high-density stacking faults, and bamboo-like segmented morphologies. Representative diameters of approximately 35–80 nm are observed in the displayed TEM images. The locally measured amorphous SiO_2_-rich surface layer decreases from approximately 2 nm at 1200 °C and 1.5 nm at 1300 °C to approximately 1.0 nm at 1400 °C.

Overall, the temperature-dependent evolution of coke from a defect-rich turbostratic structure toward a more structurally ordered turbostratic framework occurs concurrently with the nucleation and growth of SiC whiskers. The initially abundant defect sites may provide favorable reaction sites, whereas the increasingly stable carbon framework supports sustained crystal growth at higher temperatures. These results demonstrate that coke is a promising and economical internal carbon source for the in situ synthesis of SiC whiskers in refractory materials.

## Figures and Tables

**Table 1:** The XRD-derived structure parameters of coke after heat treatment at 600–1400 °C.

Temperature/°C	2θ_002_/°	d_002_/nm	FWHM/°	Lc/nm
600	25.159	0.35368	5.785	1.39
800	25.104	0.35445	5.654	1.42
1000	25.170	0.35353	5.004	1.61
1200	25.460	0.34956	3.758	2.14
1400	25.652	0.34699	2.305	3.50

**Table 2:** Raman spectral parameters of coke after heat treatment at 600–1400 °C.

Temperature/°C	D Band Position /cm^−1^	D Band FWHM/cm^−1^	G Band Position /cm^−1^	G Band FWHM/cm^−1^	I_D_/I_G_
600	1344.44	169.57	1593.65	77.19	0.71
800	1343.02	173.02	1595.33	86.23	1.02
1000	1352.25	182.85	1592.72	102.66	1.08
1200	1352.35	141.09	1594.46	85.08	1.04
1400	1348.05	117.98	1589.32	87.27	0.84

**Table 3:** Quantitative morphological parameters of SiC whiskers at different temperatures.

Temperature/°C	Average Length/μm	Average Diameter/nm	Aspect Ratio	Whisker Density/μm^−2^
1100 °C	2.5 ± 0.6	15 ± 5.5	140 ± 40	0.10 ± 0.03
1200 °C	3.4 ± 0.7	19.7 ± 8.5	173 ± 80	0.75 ± 0.15
1300 °C	4.4 ± 0.9	25.0 ± 17.0	164 ± 110	0.95 ± 0.2
1400 °C	4.0 ± 0.8	57.5 ± 31.8	70 ± 43	1.10 ± 0.2

## Data Availability

The original contributions presented in this study are included in the article. Further inquiries can be directed to the corresponding authors.
